# Mn(III) Porphyrin, MnTnBuOE-2-PyP^5+^, Commonly Known as a Mimic of Superoxide Dismutase Enzyme, Protects Cardiomyocytes from Hypoxia/Reoxygenation Induced Injury via Reducing Oxidative Stress

**DOI:** 10.3390/ijms24076159

**Published:** 2023-03-24

**Authors:** Sudha Sharma, Papori Sharma, Utsab Subedi, Susmita Bhattarai, Chloe Miller, Shrivats Manikandan, Ines Batinic-Haberle, Ivan Spasojevic, Hong Sun, Manikandan Panchatcharam, Sumitra Miriyala

**Affiliations:** 1 Department of Cellular Biology and Anatomy, Louisiana State University Health Sciences-Shreveport, Shreveport, LA 71103, USA; 2 Department of Radiation Oncology, Duke University School of Medicine, Durham, NC 27710, USA; 3 Department of Medicine, Duke University School of Medicine, Durham, NC 27710, USA; 4 Pharmacokinetics/Pharmacodynamics (PK/PD) Core Laboratory, Duke Cancer Institute, Duke University School of Medicine, Durham, NC 27710, USA

**Keywords:** superoxide, cardiomyocytes, SOD2, apoptosis

## Abstract

Myocardial ischemia-reperfusion injury (I/R) causes damage to cardiomyocytes through oxidative stress and apoptosis. We investigated the cardioprotective effects of MnTnBuOE-2-PyP^5+^ (BMX-001), a superoxide dismutase mimic, in an in vitro model of I/R injury in H9c2 cardiomyocytes. We found that BMX-001 protected against hypoxia/reoxygenation (H/R)-induced oxidative stress, as evident by a significant reduction in intracellular and mitochondrial superoxide levels. BMX-001 pre-treatment also reduced H/R-induced cardiomyocyte apoptosis, as marked by a reduction in TUNEL-positive cells. We further demonstrated that BMX-001 pre-treatment significantly improved mitochondrial function, particularly O_2_ consumption, in mouse adult cardiomyocytes subjected to H/R. BMX-001 treatment also attenuated cardiolipin peroxidation, 4-hydroxynonenal (4-HNE) level, and 4-HNE adducted proteins following H/R injury. Finally, the pre-treatment with BMX-001 improved cell viability and lactate dehydrogenase (LDH) activity in H9c2 cells following H/R injury. Our findings suggest that BMX-001 has therapeutic potential as a cardioprotective agent against oxidative stress-induced H/R damage in H9c2 cardiomyocytes.

## 1. Introduction

Myocyte injury is exacerbated by reperfusion following a period of ischemia leading to cardiomyocyte death, known as myocardial ischemia/reperfusion (I/R) injury [1]. There are several pathological mechanisms by which myocardial ischemia-reperfusion injury occurs, such as oxidative stress, mitochondrial dysfunction, apoptosis, ion accumulation, and inflammation [2,3,4]. Oxidative stress plays a vital role in myocardial ischemia-reperfusion injury, which occurs when there is an imbalance between the production of reactive oxygen species (ROS) and antioxidants. Oxidative stress signals several pathways, such as necrosis, apoptosis, and autophagy, leading to cell death. Therefore, targeting oxidative stress could be one of the therapeutic approaches to minimize ischemia-reperfusion injury [5,6]. The mitochondrion is one of the major sources of ROS during ischemia/reperfusion injury [7]. Different disease models have implied that mitochondrial production of superoxide radicals (O_2_^.−^) plays a vital role in oxidative stress [8,9]. One of the deleterious consequences of oxidative stress is lipid peroxidation and the generation of harmful aldehydes. The oxidative stress leads to peroxidation of the lipid membrane and subsequent reactive aldehyde formation, such as the formation of 4-hydroxynonenal (4-HNE) [10]. This cytotoxic aldehydes, which is highly reactive, is then able to form covalent adducts with proteins, nucleic acids, and other lipids [10]. DNA damage, fragmentation, and subsequent cell death are due to the adduction of 4-HNE to DNA [11]. 4-HNE 4HNE forms adducts with proteins via Schiff base formation and Michael addition, which involve reactions with the aldehyde group and the double bond in 4-HNE, respectively. Protein inactivation, aggregation, and change in protein conformation and function are mediated by 4-HNE–protein adducts [12]. Manganese superoxide dismutase 2 (SOD2) protects mitochondria against oxidative stress damage by dismuting superoxide into hydrogen peroxide and oxygen. Studies have shown an accumulation of 4-HNE during myocardial ischemia-reperfusion injury [13]. Therefore, targeting mitochondrial ROS and 4-HNE generation could be one of the therapeutic approaches to treat myocardial I/R injury. In this study, we used Mn(III) *meso-*tetrakis(*N*-*n*-butoxyethylpyridinium-2-yl)porphyrin, MnTnBuOE-PyP^5+^ (BMX-001) to evaluate whether H9c2 cardiomyocytes are protected from oxidative-induced damage in myocardial ischemia-reperfusion injury. In the current study, we have focused on the role of BMX-001 in scavenging free radicals and inhibiting apoptosis.

## 2. Results

### 2.1. BMX-001 Improved Cell Viability of H9c2 Cells Subjected to H/R

**Figure 1:**
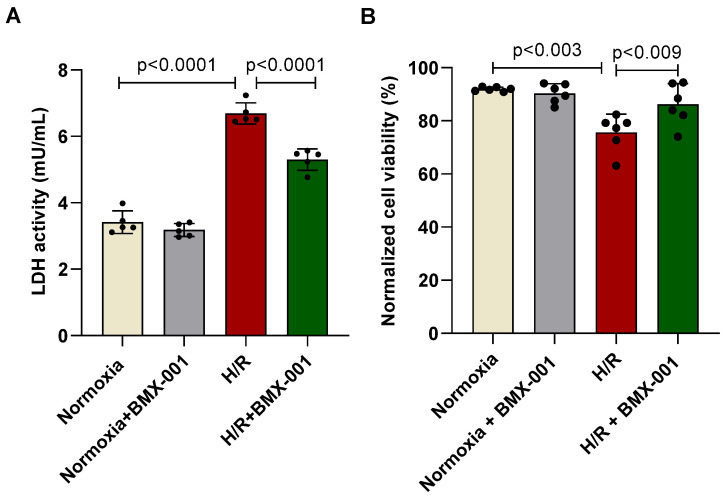
Treatment with redox regulator BMX-001 reduces H/R-induced H9c2 cardiomyocyte injury and cell death. (**A**) Following 24 h of reoxygenation, LDH activity in media was measured by assay kit. (**B**) Cell viability following 24 h of reperfusion was determined by using the trypan blue dye exclusion test. All values are mean ± SD. The *p-*value was used to determine statistical significance compared to the control group. Differences between the two groups were analyzed by the two-tailed Student’s *t*-test and of more than two groups by one-way ANOVA with post-hoc Bonferroni Multiple Comparison test.

The H9c2 cells were subjected to oxygen-glucose deprivation for 4 h followed by 24 h of reoxygenation. At first, we tested cell viability using Evans blue exclusion method following 24 h of reoxygenation. Cell viability in the H/R group was significantly lower than that of the normoxia group. Based on our previous studies with BMX-001 [14], two different concentrations of BMX-001 (10 µM and 20 μM) were tested to evaluate the cardioprotection against H/R injury in H9c2 cells. We observed that 10 µM BMX-001 pre-treatment, 24 h before undergoing H/R injury, significantly improved cell viability caused by H/R injury (Figure 1A). We thus chose 10 µM BMX-001 pre-treatment 24 h before undergoing H/R for further study. The measurement of lactate dehydrogenase, LDH activity, is a well-known marker of cardiomyocyte injury [15]. Therefore, to determine cardioprotection by BMX-001 against H/R injury, we measured LDH activity in the cultured medium. The LDH activity was significantly increased in H/R groups, while pre-treatment with BMX-001 significantly decreased LDH activity in H9c2 cells (Figure 1B).

### 2.2. BMX-001 Reduced H/R-Induced Oxidative Stress in H9c2 Cells

**Figure 2:**
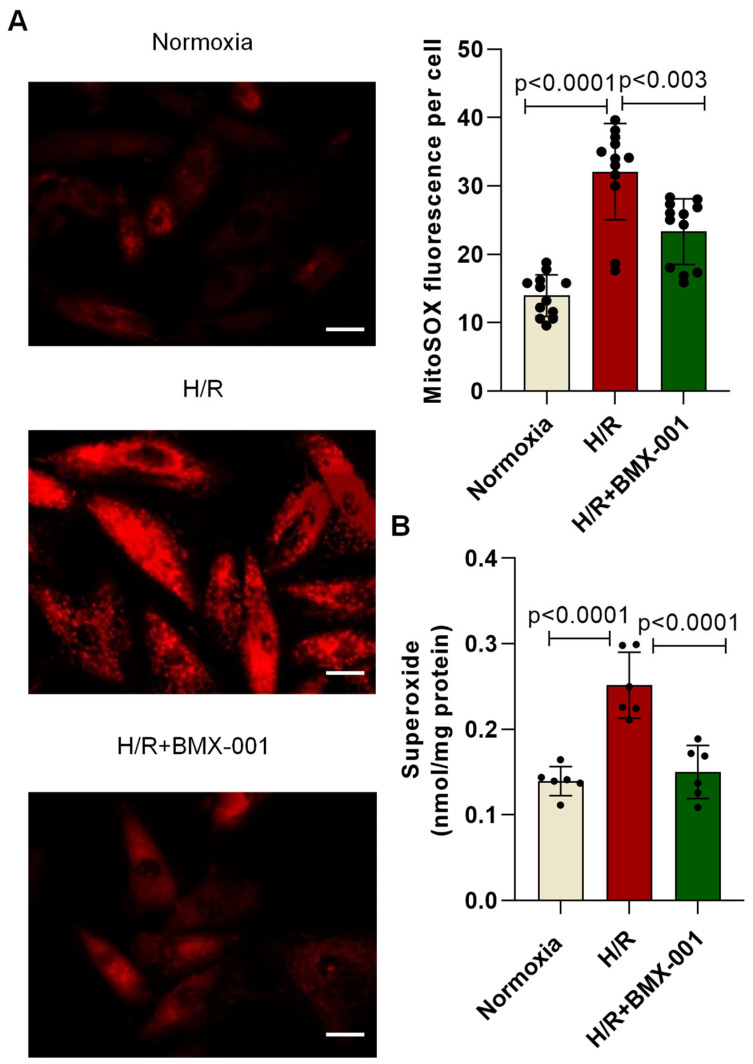
BMX-001 attenuated H/R-induced mitochondrial superoxide levels in H9c2 cells. (**A**) Mitochondrial superoxide level was determined by measuring the fluorescence of MitoSOX in H9c2 cells (magnification ×20). (**B**) Quantification of intracellular superoxide by HPLC. All values are mean ± SD. The *p*-value was used to determine statistical significance compared to the control group. Differences between the two groups were analyzed by the two-tailed Student’s *t*-test and of more than two groups by one-way ANOVA with post-hoc Bonferroni Multiple Comparison test.

Following H/R injury, significant upregulation of intracellular superoxide was observed in H9c2 cells. BMX-001 has been shown to attenuate intracellular superoxide levels following H/R injury in H9c2 cells (Figure 2A). BMX-001 is a lipophilic compound that prefers to accumulate at higher levels in mitochondria relative to the cytosol, where it dismutates superoxide in a superoxide dismutase (SOD) fashion [16]. Therefore, we tested the ability of BMX-001 to reduce the accumulation of mitochondrial superoxide following H/R injury. The mitochondrial superoxide level was largely increased in H9c2 cells following the H/R injury, which BMX-001 pre-treatment significantly attenuated (Figure 2B).

### 2.3. BMX-001 Reduces H/R-Induced Cardiolipin Peroxidation and 4-HNE Generation in H9c2 Cells

**Figure 3:**
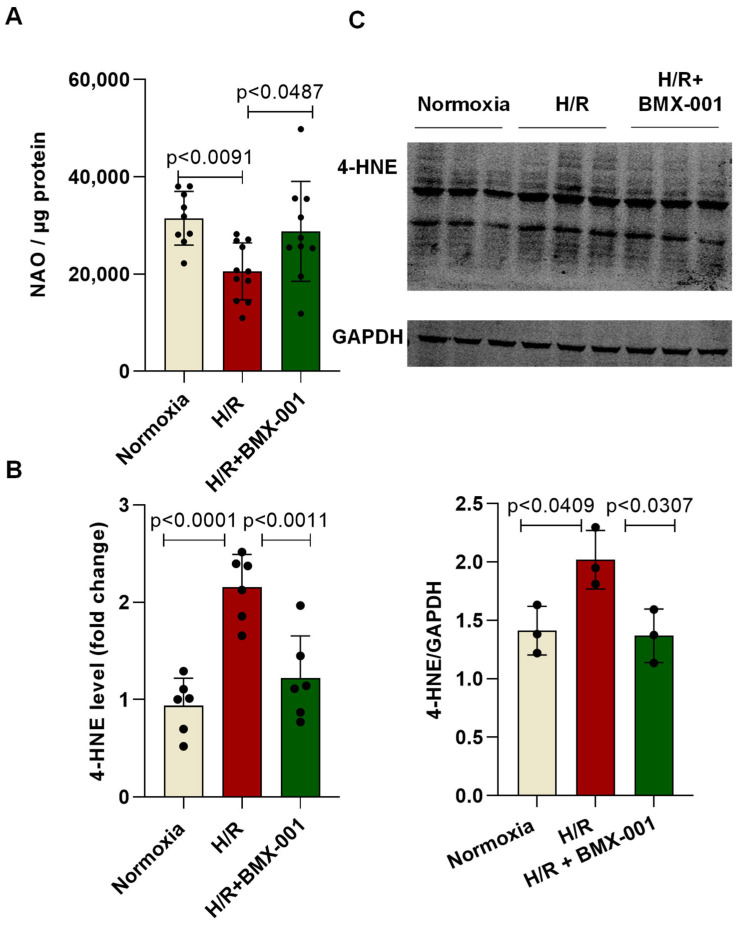
Redox regulator, BMX-001 attenuated lipid peroxidation and reactive aldehyde generation following H/R injury in H9c2 cells (**A**) Mitochondrial membrane cardiolipin peroxidation was measured by analyzing the decrease in fluorescence of 10-N-nonyl-Acridin Orange NAO (**B**) Quantification of 4-HNE by HPLC in H9c2 cell following H/R injury. (**C**) Western blot image of 4-HNE-adducted protein versus loading control, GAPDH, is shown. All values are mean ± SD. The *p-*value was used to determine statistical significance compared to the control group. Differences between the two groups were analyzed by the two-tailed Student’s *t*-test and of more than two groups by one-way ANOVA with post-hoc Bonferroni Multiple Comparison test.

One of the consequences of oxidative stress is lipid peroxidation and the generation of aldehydes which are toxic to cells. It is well-established that 4-HNE, a highly reactive and stable aldehyde, is formed as a product of cardiolipin oxidation [17]. We asked whether cardiolipin is a target of oxidation during H/R injury. Cardiolipin peroxidation was measured using 10-N-nonyl-Acridin Orange (NAO), which binds to non-oxidized mitochondrial-specific cardiolipin [18]. A decrease in the fluorescence of NAO indicates intracellular cardiolipin peroxidation [18]. Our results showed that H/R injury decreases NAO fluorescence due to the peroxidation of mitochondrial cardiolipin. BMX-001 reduced cardiolipin peroxidation in H9c2 cells following H/R injury (Figure 3A). After observing the effective reduction of cardiolipin peroxidation by BMX-001, we measured the level of 4-HNE using HPLC. We observed that the levels of 4-HNE increased during H/R injury, which was significantly attenuated by BMX-001 (Figure 3B). 4-HNE is a highly reactive aldehyde that can bind amino acids in proteins, causing modification [19]. Therefore we also measured 4-HNE protein adduction using western blot. Consistent with the data reported, we observed an increase in 4-HNE adducted protein following H/R injury [20,21]. Pre-treatment with BMX-001 significantly reduced 4-HNE adducted protein (Figure 3C).

### 2.4. Reduced Mitochondrial Bioenergetics following H/R Injury in Adult Mouse Cardiomyocytes

**Figure 4:**
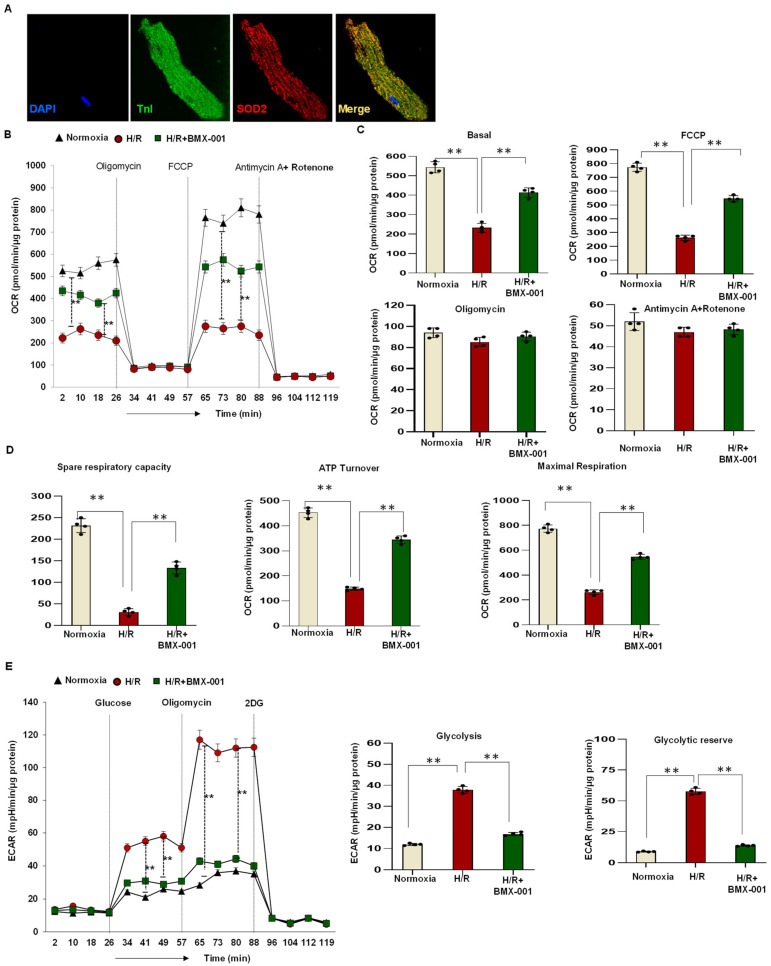
The reduced mitochondrial bioenergetics following H/R injury in the isolated adult mouse cardiomyocytes. (**A**) Isolated mouse cardiac myocytes labelled with DAPI, troponin I and SOD2 (magnification ×60) (**B**,**C**). The graph represents OCR (pmol/min/μg protein) measurements in adult mouse cardiomyocytes with Normoxia, H/R, and H/R + BMX-001 at baseline and after sequentially adding oligomycin, FCCP, and antimycin A + rotenone. (**D**) Spare respiratory capacity, ATP turnover, and maximum respiration values were analyzed in adult mouse cardiomyocytes for 4 h. (**E**) ECAR (graph and quantification) measured in adult mouse cardiomyocytes with Normoxia, H/R, and H/R + BMX-001 for 4 h represented by analyzed glycolysis and glycolytic reserve graph. All values are mean ± SD. ** *p* < 0.0001, compared to control using Student’s *t*-test for two groups and ANOVA followed by the Bonferroni post hoc test for more than two groups.

Next, mitochondrial bioenergetics were measured using an XF-24 analyzer. The isolated adult mouse cardiomyocytes (Figure 4A) were subjected to H/R injury with or without BMX-001 before oxygen consumption rate (OCR) measurement. H/R decreased OCR in cardiomyocytes, whereas BMX-001 could significantly rescue the basal OCR, spare respiratory capacity, ATP turnover, and maximal respiration (Figure 4B–D) compared with those in the H/R group. During a H/R injury, cells shift their energy production to glycolysis; the extracellular acidification rate (ECAR) measurement represents glycolysis in cells. In adult mouse cardiomyocytes subjected to H/R injury, there was a significant increase in glycolysis and glycolytic reserve, whereas pre-treatment with BMX-001 abolished this effect (Figure 4E). The considerable decrease in glycolysis and glycolytic reserve caused by BMX-001 treatment suggests that the cells were more efficient with mitochondrial energy production than those subjected to H/R injury only. Furthermore, when H9c2 cells were exposed to 4-HNE, we observed a similar rescuing effect with the BMX-001 treatment on these cardiomyocytes. These results indicate that H/R affects mitochondrial cardiomyocyte status by compromising its function.

### 2.5. BMX-001 Reduced Apoptotic Cell Death following H/R Injury in H9c2 Cells

**Figure 5:**
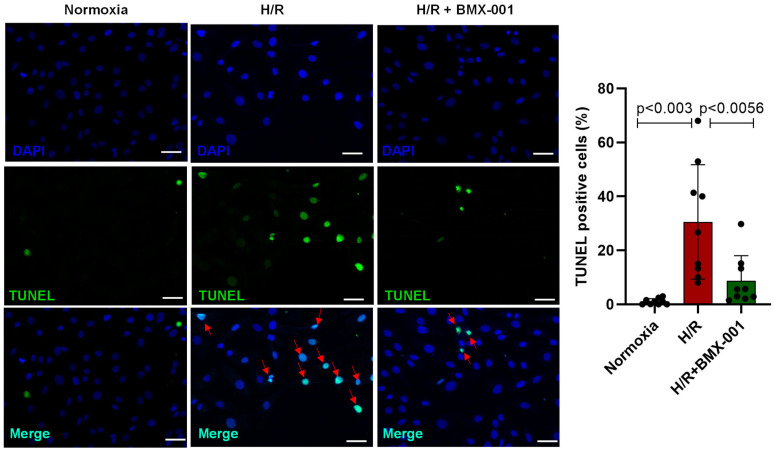
Redox regulator, BMX-001, SOD2 mimetic attenuated apoptosis following H/R injury in H9c2 cells. Apoptosis was determined by TUNEL assay (magnification ×20). The % of TUNEL-positive cells was determined by the ratio of TUNEL-positive cells to total cells. Arrow indicates apoptotic cells. All values are mean ± SD. The *p-*value was used to determine statistical significance compared to the control group. Differences between the two groups were analyzed by the two-tailed Student’s *t*-test and of more than two groups by one-way ANOVA with post-hoc Bonferroni Multiple Comparison test.

The TUNEL assay was used to evaluate the impact of BMX-001 treatment on the apoptosis of H9c2 cells following hypoxia/reoxygenation injury. Our data showed that the percentage of apoptotic cells in the H/R group was significantly higher than in the normoxia group. Furthermore, pretreatment with BMX-001 decreased the percentage rate of apoptotic cells compared to that of the cells that underwent H/R injury (Figure 5).

## 3. Discussion

Previous studies have shown that pathological conditions, such as myocardial ischemia-reperfusion injury, produce ROS and, subsequently, oxidative stress [22,23]. During myocardial ischemia/reperfusion injury, the ROS production increases through various mechanisms, such as mitochondrial respiratory chain [24], NADPH oxidases [25], and monoamine oxidases (MAOs) [26]. The electron transport chain acts as one of the primary sources of ROS during I/R injury, which increases the superoxide production within mitochondria. Therefore, targeting mitochondrial ROS is essential to provide successful therapy following myocardial ischemia/reperfusion injury. Cationic, Mn(III) *N*-substituted pyridylporphyrins are a promising class of mimetics of superoxide dismutase that scavenge superoxide radicals in enzyme-like fashion [27]. The studies also showed that cationic Mn porphyrins could impact cellular signaling pathways via their other redox activities [27]. The data on redox proteomics, as well as on oxidative stress profiling, qPCR and Western blot analyses on Nrf2 target genes on hematopoietic stem/progenitor cells and qPCR data for oxidative stress genes obtained on kidney ischemia/reperfusion rat model [28,29,30] jointly indicate that rather than scavenging superoxide directly, Mn porphyrins activate Nrf2 signaling pathways via Mn porphyrin/H_2_O_2_-driven catalysis of the oxidation of Keap1 cysteines. Such action would activate Nrf2 and upregulate endogenous antioxidative defenses, MnSOD included. While direct removal of superoxide via its dismutation in a SOD-like fashion cannot be excluded, Mn porphyrins would likely indirectly remove it via upregulation of MnSOD. The wealth of cellular and animal data has driven the progress of Mn porphyrins into five Phase II clinical trials. Four of those trials are on radio- and chemoprotection of normal tissue by BMX-001 in cancer patients bearing glioma, multiple brain metastases, anal cancer, and head and neck cancer [31]. Mn porphyrins have a beneficial effect against various diseases, including cardiovascular diseases [32,33,34]. However, the cardioprotective effect has not yet been tested in myocardial ischemia/reperfusion injury. Recently, we have shown the effect of BMX-001 on the autophagy process [14]. We overexpressed cells with Tandem fluorescent-tagged GFP-RFP LC3 to study autophagosomes and autolysosomes following rotenone treatment. Rotenone disrupted the autophagy process, but pre-treatment with BMX-001 rescued the process by equalizing the number of autolysosomes and autophagosomes. Bafilomycin, a known inhibitor of lysosomes, did not increase LC3-II expression in the rotenone-treated group, indicating that rotenone disrupted the autophagy process at the final step, which was prevented by BMX-001. There was no change in lysosomal pH between the treatment groups, indicating that the dysfunctional autophagy process was not due to lysosomal function. In this study, we used an in-vitro H9c2 cardiomyocyte hypoxia/reoxygenation model to mimic human myocardial ischemia/reperfusion injury. For the first time, this study has demonstrated that the pre-treatment of H9c2 cells with Mn porphyrin, MnTnBuOE-PyP^5+^ (BMX-001), attenuated the oxidative stress by reducing superoxide levels both within the cell and its mitochondria. In-vitro study also revealed the decrease in cardiolipin peroxidation, 4-HNE accumulation, and 4-HNE protein adduction in H9c2 cardiomyocyte pretreated with BMX-001 followed by H/R injury. Furthermore, pre-treatment with BMX-001 improved cell viability, ATP turnover, spare respiratory capacity, and reduced apoptotic cell death as shown by TUENL staining following H/R injury in H9c2 cells.

At first, we tested the ability of BMX-001 to protect against cardiomyocyte cell injury and death. LDH is a late marker of myocyte injury, reportedly elevated in circulation in myocardial ischemia/reperfusion injury [35,36]. H/R damage decreased the viable cells and increased LDH activity. However, the pre-treatment of cardiomyocytes with BMX-001 improved their viability and reduced LDH activity, indicating the protective role of BMX-001 against H/R-induced damage. Then we tested the ability of BMX-001 to reduce oxidative stress by measuring the level of superoxide at both mitochondrial and cellular levels. Consistent with previous studies, superoxide levels increased following H/R injury in H9c2 cells. Several studies have shown that cardiolipin, a unique phospholipid located almost exclusively in the inner mitochondrial membrane, is critical in maintaining protein complexes of the electron transport chain and plays an important role in mitochondrial dynamics, apoptosis, bioenergetics, protein translocation, and membrane stability [37,38,39,40]. Cardiolipins are highly susceptible to oxidation because of their unique location near the site of ROS generation and the presence of unsaturated fatty acids [39,41]. Several studies have shown that peroxidation of cardiolipin increases following ischemia/reperfusion injury [42,43]. In line with the previous results, our study demonstrated that cardiolipin peroxidation increases following H/R injury in H9c2 cardiomyocytes, and BMX-001 protects cardiolipin against oxidation. This could be due to the direct scavenging of superoxide by BMX-001. It could also be due to the indirect decrease in superoxide levels via activation of Nrf2 followed by upregulation of MnSOD. One of the major consequences of oxidative stress is lipid peroxidation and the generation of reactive aldehydes. 4-HNE is one of the reactive aldehydes formed following lipid peroxidation. 4-HNE has been subjected to extensive studies due to its ability to form adducts with nucleophilic compounds, including proteins [44]. A recent study conducted in in-vitro and in-vivo models shows that cardiolipin is a source of 4-HNE generation through cross-chain peroxyl radical addition and decomposition following an attack by free radicals [17,45]. We observed the mitigation of cardiolipin peroxidation in BMX-001- treated cells under H/R conditions. Next, we checked for the 4-HNE level by using HPLC. Our results from the present study suggest that BMX-001 decreased the level of reactive aldehyde, 4-HNE, under the H/R condition in our in-vitro model. H/R injury-induced oxidative damage elicits a mitochondrial stress response in the cardiomyocytes triggering mitochondrial impairment. The mitochondrial bioenergetics was diminished following H/R injury in isolated adult mouse cardiomyocytes and when H9c2 cardiomyocytes were exposed to 4-HNE. H/R mediated oxidative stress or exposure to 4-HNE, lowered basal OCR, and decreased spare respiratory capacity, ATP turnover, and maximal respiration in these cells. When treated with BMX-001, the mitochondrial bioenergetic profile was significantly revived. The glycolysis and glycolytic reserve measured with ECAR was increased following H/R injury, but BMX-001 treatment halted the trend towards an increase in glycolysis. These findings suggest that inhibition of oxidative stress limits the mitochondrial damage caused by H/R injury. Cardiomyocyte apoptosis following myocardial ischemia/reperfusion injury mediates myocardial cell loss, thereby subsequently increasing the incidence of myocardial dysfunction, ventricular remodeling, and even heart failure [46,47,48]. Blocking apoptosis has been shown to protect against cardiomyocyte death [49,50,51]. Mitochondria play a central role in apoptosis characterized by the breakage of DNA [52,53]. In the present study, apoptosis was determined by TUNEL assay, and BMX-001 was been shown to protect H9c2 cells from injury and death by reducing apoptosis. While our study presents novel insights into the potential of BMX-100 as an effective agent for mitigating oxidative stress in vitro, we acknowledge the limitations associated with the absence of relevant clinical models in our experimental design. To further strengthen the novelty and clinical relevance of our findings, future research should explore the efficacy of BMX-100 in relevant clinical models of oxidative stress, such as in vivo animal models and human clinical trials. Such studies will help establish the specific clinical relevance of our findings and provide a more comprehensive understanding of BMX-100 as a therapeutic agent for the treatment of oxidative stress-related diseases.

## 4. Methods and Materials

### 4.1. In-Vitro Model of Hypoxia/Reoxygenation Injury

H9C2 cells (American Type Culture Collection (ATCC), Manassas, VA, USA) were cultured in Dulbecco’s modified Eagle medium (Fisher Scientific, Waltham, MA, USA) supplemented with 10% fetal bovine serum and 1% penicillin/streptomycin. The culture environment was 37 °C in an incubator with 5% CO_2_ and normal ambient oxygen levels. Whereas to isolate adult cardiomyocytes, the hearts of 8–12-week-old C57/BL6J mice were exposed by opening the chest under anesthesia to obtain highly pure myocyte fractions. The descending aorta was cut, and the heart was immediately flushed with 7 mL EDTA buffer injected into the right ventricle. Subsequently, the ascending aorta was clamped, and the heart was transferred to a 60-mm dish with fresh EDTA buffer. Sequential injection of 10 mL EDTA buffer, 3 mL perfusion buffer, and 30 to 50 mL collagenase buffer into the left ventricle allowed for the digestion of the heart tissue. The constituent chambers, including the atria, LV, and right ventricle, were then separated and gently pulled into 1-mm pieces with forceps. Hearts were digested on average for 3–4 min. Then the collagenase solution was flushed from the tissue with Ca^2+^-free Tyrode solution. Cells were filtered through a nylon mesh and allowed to settle by gravity for 5 min. [Ca^2+^] in the buffer was increased stepwise, from 0.6 μM, 0.2 mM, 0.6 mM to 1.2 mM, by suspending the cell pellet in modified Krebs–Henseleit buffer (in mM: 0.5 EDTA, 5.1 KCl, 0.6 MgSO_4_, 118 NaCl, 1.2 KH_2_PO_4_, 10 glucose, 1 NaHCO_3_, 10 HEPES, 2 mg/mL BSA Fraction V, Sigma-Aldrich, St. Louis, MO, USA). Cells were always allowed to settle by gravity. The myocyte-enriched cell pellet from each round produced a highly pure myocyte fraction. The relative number of rod-shaped and rounded cells were determined by transferring 2 mL of the cell suspension to a 35 mm culture dish and counting three fields of view at 10× magnification. The adult cardiac myocytes were then resuspended in prewarmed plating media and plated onto laminin (5 μg/mL), Thermo Fisher Scientific, Waltham, MA USA precoated tissue culture plastic at an application-dependent density in a humidified tissue culture incubator (37 °C, 5% CO_2_) for further experimentation. For hypoxia/reoxygenation injury, the cells were first grown in a glucose-free medium without fetal bovine serum and then placed in a chamber. Next, N_2_ gas was flown in a chamber for 6 min to replace oxygen. Then the cells were reoxygenated for 4 h by changing the initial media with complete high glucose DMEM medium for 24 h in a normal CO_2_ incubator.

### 4.2. Cell Viability Assay

The trypan blue exclusion method measured the viability of H9c2 cells after 24 h of reperfusion. Cells with or without treatments with BMX-001 were resuspended with media. Then, the 0.4% trypan blue solution (Sigma-Aldrich, St. Louis, MO, USA ) was added in a 1:1 ratio. The cell suspension was added to Neubauer’s chamber and counted under a bright field microscope.

### 4.3. Lactate Dehydrogenase Activity Assay

Lactate dehydrogenase activity assay was measured in the media following reperfusion according to the protocol given by the manufacturer (Abcam, Cambridge, MA, USA, ab102526). After pre-treatment with BMX-001, cells were exposed to 4 h of hypoxia and then 24 h of reperfusion. The reaction mixture was prepared for standard and sample wells according to the instructions given by the manufacturer (Abcam, Cambridge, MA, USA, ab102526). Briefly, after adding 50 µL of the reaction mixture, wells were mixed. Absorbance was measured as molar absorptivity at 450 nm on a ClarioStar (BMG LABTECH, Cary, NC, USA) in a kinetic mode every 2 min for 60 min at 37 °C in dark.

### 4.4. Measurement of Superoxide

For intracellular superoxide measurement, the fluorescent probe DHE was used at a concentration of 10 µM. Cells were kept in DHE for 10 min in the incubator. Following incubation, cells were washed with PBS. The cell lysate was further processed according to previous protocols [54] and was measured by HPLC analysis coupled with UV/vis and fluorescence.

### 4.5. Mitochondrial Superoxide Measurement

Mitochondrial superoxide was measured using a fluorescent probe MitoSOX (Thermo Fisher Scientific, Waltham, MA, USA) diluted to a final concentration of 5 µM. Cells were incubated in MitoSOX in normal media for 15 min in a normal CO_2_ incubator. Following incubation, cells were washed and imaged under a fluorescent microscope.

### 4.6. Measurement of Cardiolipin

NAO 0-N-nonyl-Acridin Orange, (Molecular Probes, Inc., Eugene, OR, USA) was used to measure cardiolipin peroxidation. NAO specifically binds to non-oxidized cardiolipin, thereby decreasing its fluorescence—the indicator of cardiolipin peroxidation [18]. In this study, H9c2 cells were seeded at 96 well plates and treated with or without BMX-001 for 24 h before undergoing H/R injury. Following 24 h of reperfusion, cells were treated with 10 µM NAO and incubated for 20 min. After incubation, cells were washed twice, and fluorescence was measured at 530 nm–excitation and emission at 485 nm in CLARIOstar (BMG LABTECH, Cary, NC, USA).

### 4.7. Western Blot Analysis

H9c2 cells were pretreated with or without BMX-001 for 24 h before undergoing hypoxia/reoxygenation injury. After 24 h of reperfusion, cell lysates were prepared in ice-cold RIPA lysis buffer (Pierce Ripa buffer, ThermoFisher, Waltham, MA, USA). Protein concentration was measured by using BCA BCA Protein Assay Kit (Abcam, Cambridge, MA, USA).

### 4.8. Mitochondrial Bioenergetics

As the state of the mitochondria reflects the physiological state of the cells, mitochondrial bioenergetics were measured in isolated adult cardiomyocytes and H9c2 cells [55]. The OCR (oxygen consumption rate) and ECAR (extracellular acidification rate) were analyzed using a Seahorse extracellular flux analyzer (XF-24, Seahorse Biosciences, Chicopee, MA, USA). Isolated adult cardiomyocytes [14] or H9c2 cells in a Seahorse plate subjected to H/R injury or with 4-HNE for cell mitochondrial bioenergetics analysis. BMX-001 was added 3 h before the experimental setup. The cells’ real-time bioenergetic activity and treatments’ effects were observed as free protons, and the oxygen concentration was measured using the XF-24. OCR and ECAR values were quantified in pmol/min/μg and mpH/min/μg, respectively, with normalization for the total protein content [56]. The initial basal value of OCR is interrupted by the addition of oligomycin (Complex V inhibitor), giving values for ATP-linked OCR. FCCP (an uncoupler) and rotenone + antimycin (Complex I and Complex III inhibitor) addition determines the maximal OCR capacity and spare OCR capacity, respectively. For ECAR experiments, a glucose-free medium was used. Following the sequential addition of glucose (25 mM), oligomycin (1 μg/mL), and deoxyglucose (25 mM), we measured the rate of glycolysis and glycolytic reserve in these cells [54,57].

### 4.9. H9c2 Cells TUNEL Assay

The H9c2 cells underwent H/R injury during 24 h of treatment with or without BMX-001. The number of apoptotic cells was measured by using In Situ Cell Death Detection Kit (#12156792910, Roche, Indianapolis, IN, USA). Briefly, 50 µL of TUNEL mixture was added to cells and incubated at 37 °C in the dark humified chamber for 60 min. Following incubation, cells were washed with PBS three times. The amount of TUNEL-positive cells was analyzed under a fluorescent microscope after staining of the nucleus with DAPI. The percentage of the TUNEL-positive cells was calculated as the: TUNEL-positive cells per field/total cells per field.

### 4.10. Statistical Analysis

Statistical analysis was performed using Prism 8.0 (GraphPad Prism, version 8.4.2, San Diego, CA, USA, RRID: SCR_002798). Data are reported as means ± standard deviation (SD), with all experiments carried out with a minimum of three replicates. One-way analysis of variance (ANOVA) followed by the Bonferroni post hoc test or the unpaired Student’s *t*-test (Mann-Whitney U test: non-parametric *t*-test to compare whether there is a difference in the dependent variable for two independent groups) was used to identify significant differences between groups. A *p*-value of less than 0.05 was considered significant.

## 5. Conclusions

Our study demonstrated that H/R injury-induced cell death and cardiomyocyte injury were mitigated by the treatment with BMX-001. Hypoxia reoxygenation injury in H9c2 cells elevated oxidative stress, followed by the increase in cardiolipin peroxidation and 4-HNE generation leading to subsequent 4-HNE protein adduction. However, treatment with BMX-001 reduced cardiolipin peroxidation, 4-HNE generation, and 4-HNE adducted protein levels. In this study, we also demonstrated that BMX-001 suppressed oxidative stress under H/R conditions, reducing superoxide at both mitochondrial and cellular levels. Further, BMX-001 reduced the number of dead cells by suppressing apoptosis. This study provides insight into the cardioprotective potential of BMX-001 to mitigate I/R-induced damage of cardiomyocytes. Our findings suggest that BMX-100 may be a promising candidate for further investigation in clinical trials for the treatment of oxidative stress-related diseases.

## Data Availability

Not applicable.
